# Analysis of Spatiotemporal Urine Protein Dynamics to Identify New Biomarkers for Sepsis-Induced Acute Kidney Injury

**DOI:** 10.3389/fphys.2020.00139

**Published:** 2020-03-03

**Authors:** Yiming Li, Junke Long, Jiaquan Chen, Jing Zhang, Yi Qin, Yanjun Zhong, Fen Liu, Zhiyong Peng

**Affiliations:** ^1^Department of Critical Care Medicine, Zhongnan Hospital of Wuhan University, Wuhan, China; ^2^Department of Cardiovascular Medicine, The Second Xiangya Hospital, Central South University, Changsha, China; ^3^Department of Critical Care Medicine, The First Affiliated Hospital of Nanchang University, Nanchang, China; ^4^ICU Center, The Second Xiangya Hospital, Central South University, Furong, China; ^5^Center of Critical Care Nephrology, Department of Critical Care Medicine, University of Pittsburgh School of Medicine, Pittsburgh, PA, United States

**Keywords:** sepsis-induced acute kidney injury, mass spectrometry, proteomics, biomarker, early diagnose

## Abstract

Acute kidney injury (AKI) is a frequent complication of sepsis and contributes to increased mortality. Discovery of reliable biomarkers could enable identification of individuals with high AKI risk as well as early AKI detection and AKI progression monitoring. However, the current methods are insensitive and non-specific. This study aimed to identify new biomarkers through label-free mass spectrometry (MS) analysis of a sepsis model induced by cecal ligation and puncture (CLP). Urine samples were collected from septic rats at 0, 3, 6, 12, 24, and 48 h. Protein isolated from urine was subjected to MS. Immunoregulatory biological processes, including immunoglobin production and wounding and defense responses, were upregulated at early time points. Kyoto Encyclopedia of Genes and Genomes (KEGG) pathway enrichment analyses identified 77 significantly changed pathways. We further examined the consistently differentially expressed proteins to seek biomarkers that can be used for early diagnosis. Notably, the expression of PARK7 and CDH16 were changed in a continuous manner and related to the level of Scr in urine from patients. Therefore, PARK7 and CDH16 were confirmed to be novel biomarkers after validation in sepsis human patients. In summary, our study analyzed the proteomics of AKI at multiple time points, elucidated the related biological processes, and identified novel biomarkers for early diagnosis of sepsis-induced AKI, and our findings provide a theoretical basis for further research on the molecular mechanisms.

## Highlights

- New biomarkers were identified by mass spectrometry analysis in sepsis-induced AKI.
- Proteomics dynamic changes were exhibited at multiple time points.
- Immunoregulatory biological processes were upregulated at early time point.
- PARK7 and CDH16 could accurately discern sepsis-induced AKI patients with high AUC.

## Introduction

Sepsis is associated with up to 50% of AKIs, and up to 60% of septic patients have AKI complications (Uchino et al., 2005; Bagshaw et al., 2009). The morbidity of AKI was found to be associated with the severity of sepsis in a cohort study of 315 patients (Lopes et al., 2009). Because AKI has a high incidence and is always associated with poor outcomes, development of new approaches for the prevention and early diagnosis of AKI is of great importance (Vanmassenhove et al., 2017). In addition, several notable drug trial failures raised concerns that AKI was not diagnosed early enough for effective intervention and that a rise in serum creatinine itself is not a sensitive enough marker (Griffin et al., 2018). Biomarkers have been proven to be important tools for current and future AKI research and clinical management (Griffin et al., 2019). Novel biomarkers can not only enable assessment of declines in kidney function but also indicate structural damage in the kidneys at earlier time points than increases in serum creatinine. Early diagnosis of sepsis-induced AKI will enable appropriate and timely treatment, which may contribute to better outcomes of AKI (Bagshaw et al., 2009). Researchers have been searching for new biomarkers to diagnose AKI with a novel detection technology. Thus, new biomarkers with the higher AUC values than current biomarkers are expected to benefit the early diagnosis of AKI (Bagshaw et al., 2009). The search for biomarkers associated with early diagnosis will aid in solving the clinical problems of sepsis-induced AKI.

Protein quantitation is an important method for the identification of new biomarkers, and MS combined with liquid chromatography is becoming a crucial tool in both biological and clinical research settings (Prasad et al., 2017). Liquid chromatography-tandem mass spectrometry (LC-MS/MS) is a powerful technology suited for a wide range of applications, including protein and peptide biomarker analysis in clinical chemistry (Grebe and Singh, 2016). Due to its straightforward method development for a wide variety of candidate analytes, LC-MS can often represent a more precise alternative to immunobinding assays and biomarker panel analyses in the field of targeted proteomics (Himmelsbach, 2012; Rauh, 2012). The use of unlabeled protein profiles to examine differentially expressed proteins in urine samples largely eliminates the variations and biases in replicate MS measurements (Atrih et al., 2014; Montoya et al., 2019). Unlike blood samples, urine samples can be collected non-invasively; moreover, the protein compositions of urine samples are relatively simple, stable and easier to analyze (Guo et al., 2018).

Given the benefits offered by LC-MS methodology, the purpose of this study was to identify and validate early biomarkers of sepsis-induced AKI using label-free proteomics, thus providing the possibility of early diagnosis. Bioinformatic analysis of differentially expressed proteins suggested the involvement of these proteins in inflammation, immune responses, and metabolic regulation. In addition, we found new, specific biomarkers that can predict septic AKI. Our work will have major impacts on the early prediction of and intervention for septic AKI.

## Materials and Methods

### Animal Model of AKI

Cecal ligation and puncture surgery was performed to induce sepsis as previously described (Rittirsch et al., 2009). This model was modified based on the ligated percentage of the cecum, the number of punctures and contents of squeezed feces to induce AKI (Peng et al., 2014). We followed the methods of Li et al. (2019). Briefly, 10–12 weeks old male Sprague-Dawley rats (400–500 *g*) were anesthetized with isoflurane, and a midline incision (2 cm) was made below the diaphragm to expose the cecum. Thirty percent of the cecum was ligated at the colon juncture with a 3–0 silk ligature suture, punctured twice with an 18-gauge needle, and laced back in the abdomen; then, the incision was closed in two layers. Sham surgery was performed in the same way as the CLP surgery but without ligation and puncture. Rats were fluid-resuscitated with 5 ml per 100 g saline injected subcutaneously.

### Collection of Rats’ Urine and Blood

Metabolic cages (Nalgene, Thermo Fisher) were used for collecting urine. Metabolic cages consist of a circular upper portion, which houses the rat, and a lower collection chamber with a specialized funnel that separates fecal pellets and urine that fall through the grid floor for their collection into 2 separate tubes. Rat urine samples were collected at 0, 3, 6, 12, 24, and 48 h after CLP surgery and preserved at −80°C for proteomic analysis. Blood (0.4 ml) was drawn from a femoral vein catheter at indicated time after CLP. We collect the blood with a heparin-coated syringe. Heparinized blood was centrifuged at 10,000 × *g* for 10 min to separate the plasma, which was collected for serum creatinine detection. All experiments were performed in accordance with Chinese legislation on the use and care of laboratory animals and were approved by the Animal Care and Use Committee of Nanchang University.

### Evaluation of the Renal Function

The serum concentration of creatinine was measured using commercial kit reagents (Institute of Jiancheng Bioengineering, Nanjing, China). The absorbance was detected by Thermo Scientific Microplate Reader.

### Sample Preparation for MS

Two milliliters of urine per sample (18 samples) was centrifuged at 2,000 × *g* for 10 min at 4°C, and 10 KDa ultrafiltration tubes were used to filter the samples. The protein supernatant was mixed with 200 μL of 8 M urea in Tris–HCl and centrifuged at 14,000 × *g* for 15 min. Then, 10 μL of 10 X IAA in urea solution was added to the concentrate in the filter. The spin filter was incubated and centrifuged. Then, 0.1 μg/μL of LysC was added. Following incubation, 40 μL of 100 mM ABC solution was added and centrifuged at 14,000 × *g* for 10 min and repeated 1X to increase peptide yield. Finally, 50 μL of 0.5 M NaCl solution was added to the spin filter and centrifuged. Following the first digestion, spin filters were washed. Peptides were eluted, acidified with TFA, and desalted on a C18 MacroSpin column (The Nest Group, Southboro, MA, United States). The concentration of the peptides was determined using a microplate colorimetric assay (Bio-Rad).

### Urinary Protein Analysis by LC−MS

The total peptide concentrations were measured by NanoPhotometer (Implen, Munich, Germany). The peptide mixtures were loaded into a reverse−phase microcapillary column (0.1 × 150 mm, packed with Magic C18, 100 μm, 100Å; Michrom Bioresources). The eluted peptides were analyzed using the Orbitrap Fusion Lumos MS system (Thermo Fisher Scientific). The instrument was run with peptide recognition mode enabled. The full MS scans were acquired at a resolution of 120,000 at m/z 200 and 60,000 at m/z 200 for MS/MS scan. The maximum injection time was set to 500 ms for MS and 50 ms for MS/MS. The normalized collision energy was 27, and the isolation window was set to 1.6 Th. The dynamic exclusion duration was 60 s. Each sample was analyzed at least three times.

### Database Searching and Protein Identification

The raw data files were searched using MaxQuant 1.5.2.8 (Cox et al., 2014). MS data were searched against the UniProt *Rattus norvegicus* reference proteome. All proteins with a cutoff below a 1% false discovery rate were exported to ensure only high-confidence protein identifications. Label-free quantification was carried out in MaxQuant using the intensity determination and normalization algorithm as previously described (Cox et al., 2014). The quantitative protein ratios were weighted and normalized by the median ratio in MaxQuant software. All MS-proteomics data have been made publicly available via iProX with the dataset identifier IPX0002025000.

### Patient Recruitment and Urine Collection

Ethical approval was granted by the institutional Research Ethics Committee at the Zhongnan Hospital of Wuhan University (NO. 2017004). All patients provided informed written consent to participate in the study. The urine was collected at 12 h after sepsis patients were diagnosed with AKI from Kidney Disease: Improving Global Outcomes (KDIGO) stage 1 (serum creatinine 1.5 times the baseline level or ≥0.3 mg/dl (≥26.5 μmol/l) urine output increase <0.5 ml/kg/h for 6 to 12 h). The urine samples were collected from patients with or without acute kidney injury.

### ELISA

KLK1, CLUSTERIN, HASPA5, AXL, CDH16, CFH, NAPSA, PARK7 and PGLYRP2 levels in human urine were measured by Elisa kit (Bio-Swamp Life Science, Shanghai, China).

### Association of Clinical and Pathological Characteristics With Differentially Expressed Protein in Sepsis-Induced AKI

Receiver operating characteristic curve analysis was performed to evaluate the characteristic difference in PARK7 and CDH16 expression among urine from normal individuals and patients with sepsis-induced AKI. SPSS software was used to draw the ROC curves and calculate the sensitivity, specificity, and AUC values.

#### Statistical Analysis

The data were analyzed with SPSS 25.0 software (SPSS Inc., Chicago, IL, United States) and GraphPad Prism 8.0 (San Diego, CA, United States). To compare the differences among the groups, a one-way analysis of variance (ANOVA) was used for normally distributed data, whereas the Kruskal–Wallis test was used for non-normally distributed data. To compare the difference between the two groups, an independent *t*-test was used for normally distributed data, whereas the Mann–Whitney *U*-test was used for non-normally distributed data. A *p* value < 0.05 indicated a significant difference.

## Results

### The Workflow in Proteomics Analysis

**FIGURE 1:**
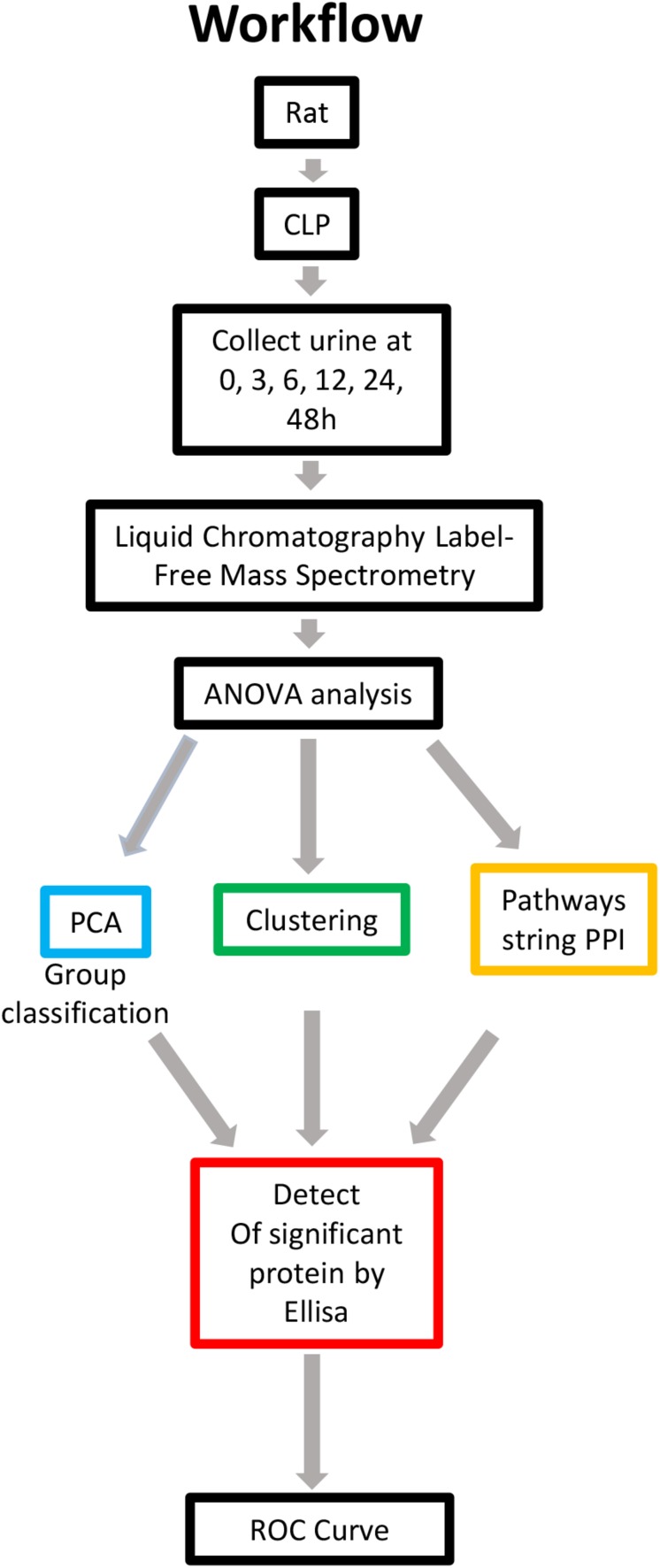
Overview of methods employed and results obtained. Rat urine was collected at 0, 3, 6, 12, 24, and 48 h and subjected to label-free MS. One-way ANOVA was performed with a *p* value cutoff of 0.05. For group classification, PCA, hierarchical clustering analysis and PPI were used, and significant proteins were identified. Furthermore, validation was performed on selected proteins in human urine. AUC and a diagnostic cutoff value of differential expression protein were calculated by ROC curve. ANOVA, analysis of variance; MS, mass spectrometry; PCA, principal component analysis; PPI, protein-protein interaction (via OmicsNet and STRING). AUC, area under curve; ROC, receiver operating characteristic.

Rats were subjected to CLP to induce sepsis-AKI. The urine and blood of each rat were collected at 0, 3, 6, 12, 24, and 48 h. Serum creatinine was detected to determine the severity of kidney injury. Kidney injury was defined according to KDIGO SCr criteria (Kellum et al., 2013). Urine samples in which serum creatinine levels from 6h after CLP increased ≥ 1.5 times from baseline were selected and subjected to label-free MS. Serum creatinine levels in each rat are shown in Supplementary Table S1. Since the urine output of each rat was scant, a urine sample of same time point after CLP was pooled into one pool as one sample. There were three samples at each time point. Proteomics using label-free strategy in 18 urine samples was analyzed by different methodologies. The data quality control results demonstrated that the whole analytical process was stable and reliable (Supplementary Figures S1A,B). Application of a 2-dimensional LC-MS strategy using isobaric tags for relative and absolute quantitation (iTRAQ) reagents identified a total of 1,258 unique rat proteins. One-way ANOVA was used to identify the differentially expressed proteins. We additionally performed a comparison of PPIs at different time points to analyze the altered pathways. Differentially expressed proteins with a continuous change that began from the onset of kidney injury and lasted for the whole course of the disease were selected as the biomarkers for early identification and diagnosis of AKI. Urine samples from 30 patients with sepsis-induced AKI and 59 sepsis patients without AKI were used for ELISA. The workflow is visualized in Figure 1.

### Principal Component Analysis and PPI Analysis in Urine From Sepsis-Induced AKI

**FIGURE 2:**
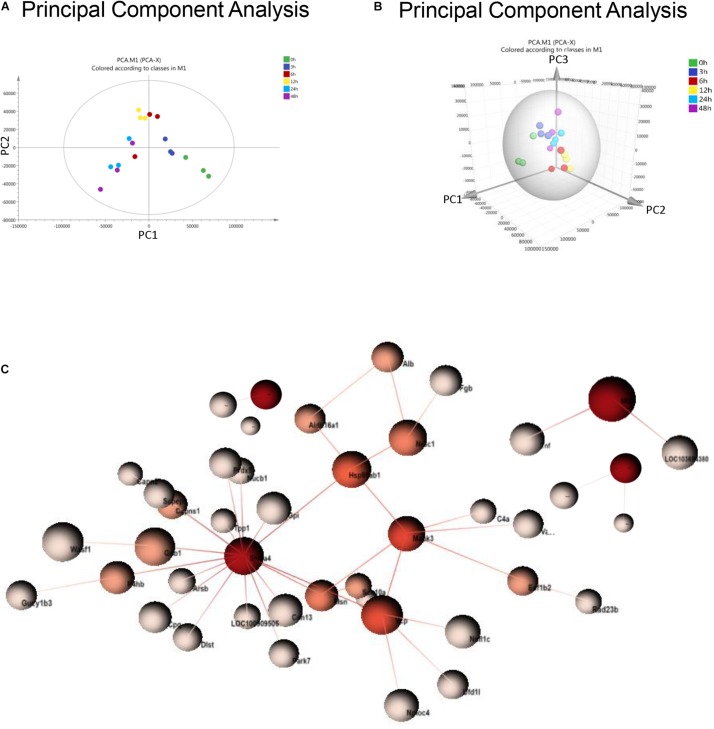
PCA and PPI of urine proteomics. **(A)** PCA of urine proteomics. The PCA two-dimensional scatter plot represents the differential protein expression patterns of urine after sepsis. Axis: *X* = PC1: PCA Component 1 (48% variance); *Y* = PC2: PCA Component 2 (27.4% variance). **(B)** PCA were shown in the 3-dimensional figure. **(C)** PPI between differentially expressed proteins was analyzed by OmicsNet.

Principal component analysis is a statistical technique for simplifying datasets and determining principal components (PCs) that best explain variation among samples. To obtain a global protein profile, PCA score plots were first applied to display the trends of the samples in the control (0 h) and sepsis-induced AKI groups. The PCA model was based on two principal components that summarized 75.4% of the variation in the dataset in total (Figure 2A). The score plots revealed that the control rats (0 h) and the rats with kidney injury were obviously separated. There was a clear discrimination of the positive fractions (0 and 3 h) from the negative fractions (12, 24, and 48 h) by PC1, which contributed to most of the variance (48%). In addition, from 0 to 12 h, the principal components of urine were changed according to PC1, and from 12 to 48 h, the variation was accounted for by PC2 to a large extent. PC2 contributed to 27.4% of the variance. PC3 contributed to only 5.17% of the variance (Figure 2B). PCA showed remarkable differences among the six groups, demonstrating that sepsis substantially influenced the urinary protein profiles.

One-way ANOVA was performed to determine the differentially expressed proteins. There were 377 differentially expressed proteins in urine after sepsis-induced AKI (Supplementary Table S2). PPI network analysis of these proteins was performed to further identify key proteins and peptides associated with sepsis-induced AKI. The PPI network was composed of 49 uniquely expressed nodes and 43 edges. Slc2a4 and Mapk3 interact more with other proteins (Figure 2C).

### Protein Profile Changed During Sepsis-Induced Kidney Injury

**FIGURE 3:**
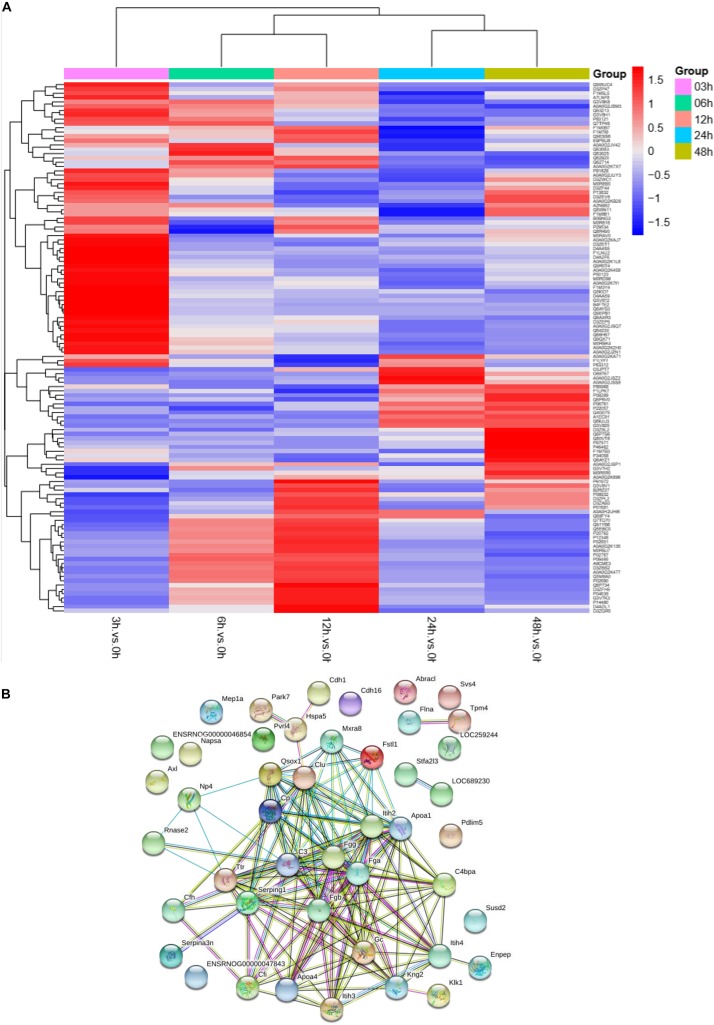
The protein profile changed during sepsis-AKI. **(A)** Heatmap showed the differential expression protein in urine at the indicated time (3, 6, 12, 24, and 48 h) compared with 0 h analyzed by one-way ANOVA. The fold change was defined as ≥1.5 or ≤0.5. **(B)** PPI networks of the differentially expressed proteins were constructed based on the STRING database web tool.

To understand the change in the protein profile with time, one-way ANOVA was performed. The fold change was defined as ≥1.5 or ≤0.5. There were 123 differentially expressed proteins in urine after sepsis-induced AKI. Heatmap analysis showed the differentially expressed protein levels in the urine at the indicated times (3, 6, 12, 24, and 48 h) compared with the levels at the baseline (0 h) (Figure 3A).

**FIGURE 4:**
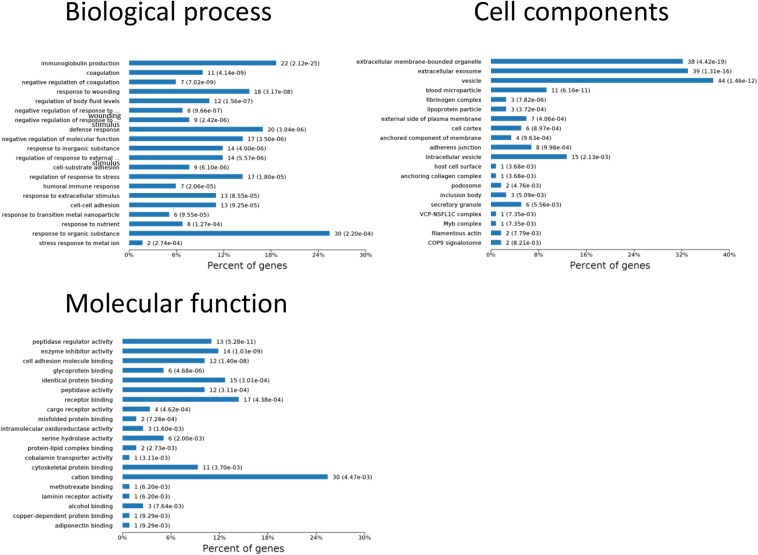
Biological processes, cell components, and molecular functions in which differentially expressed proteins are involved. *P* < 0.05.

One aim of our study was to gain insight into the pathways and networks altered in the urine from the onset of sepsis-induced AKI. Based on the STRING database web tool, PPI networks of the differentially expressed proteins were constructed (Figure 3B). The PPI network featured 45 uniquely expressed nodes and 147 edges. The biological process, cell components and molecular function in which differentially expressed proteins were involved are shown in Figure 4. The immunoregulatory biological processes, including immunoglobin production, response to wounding and defense response, were significantly changed. A vesicle, extracellular exosome, and extracellular membrane-bound, organelle-associated proteins were excreted into the urine after AKI. The Gene Ontology (GO) analyses of each time point are shown individually in Supplementary Figures S2–S4. In the biological process (Supplementary Figure S2), the protein regulating metabolic process was the earliest altered in the first 3 h of sepsis-induced AKI, and subsequently (6 h later), immunoglobulin production was increased significantly. From 6 to 24 h, the differentially expressed protein profiles involved in the biological process changed slightly. Moreover, responses to organic substances, wounding, and stimulus were the leading biological processes. Extracellular exosomes, extracellular membrane-bound organelles, and vesicles were the three leading altered cell components during the 48 h after septic AKI (Supplementary Figure S3). The proteins related to the cell function of peptidase regulator activity, enzyme inhibitor activity, and peptidase activity increased in the early 6 h and remained at a high level of expression (Supplementary Figure S4).

### Kyoto Encyclopedia of Genes and Genomes (KEGG) Enrichment Analysis

**FIGURE 5:**
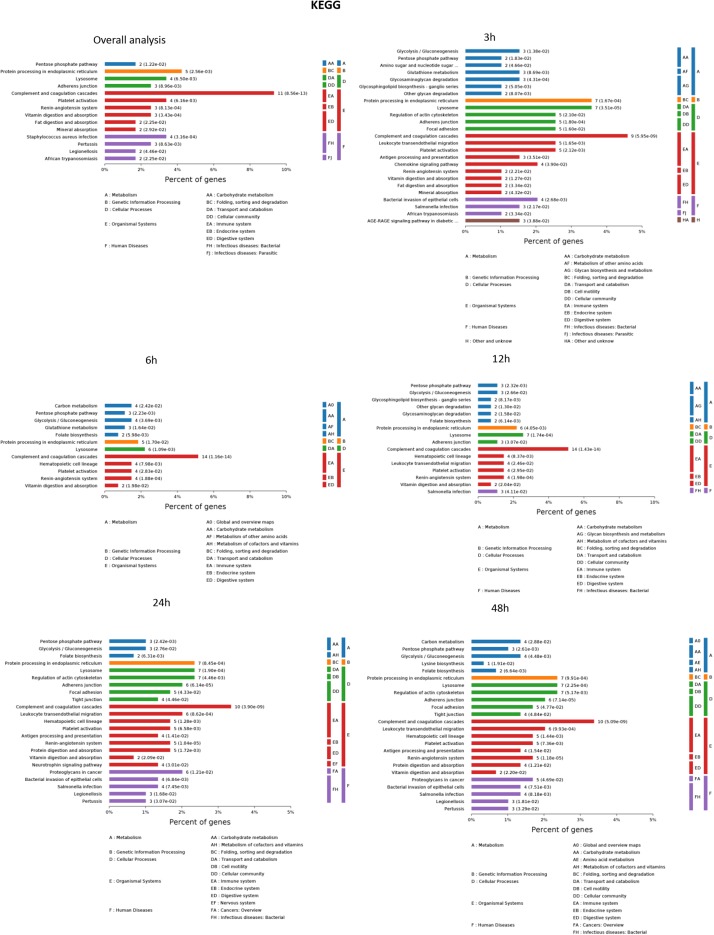
The KEGG enrichment analysis of significantly changed pathways overall and at the indicated time (3, 6, 12, 24, and 48 h) compared with 0 h. *P* < 0.05.

The results of significantly enriched KEGG analysis in the first 48 h of the AKI pathophysiological process are shown in Figure 5. KEGG pathway enrichment analysis from 77 significantly changed pathways showed that these genes were mainly involved in the complement and coagulation cascades, *Staphylococcus aureus* infections, protein processing in the endoplasmic reticulum, and platelet activation. The pathways were dramatically changed at 3 h after CLP. The immune system and metabolic process changed most significantly. KEGG enrichment analysis revealed that the organismal system, including the immune system, endocrine system, and digestive system, remained the same in 12 h as in 6 h. Classes of the pathway were decreased in the urine sample at 6 h after sepsis-induced AKI. The significantly changed pathways remained stable from 24 h onward. The immune system was changed throughout the pathological process of AKI, which influences and interacts with other cell processes, indicating that the inflammation pathways play a central role in the pathophysiology of sepsis-induced AKI.

### Protein Profile Changes According to Four Continuous Changing Models in Sepsis-Induced AKI

**FIGURE 6:**
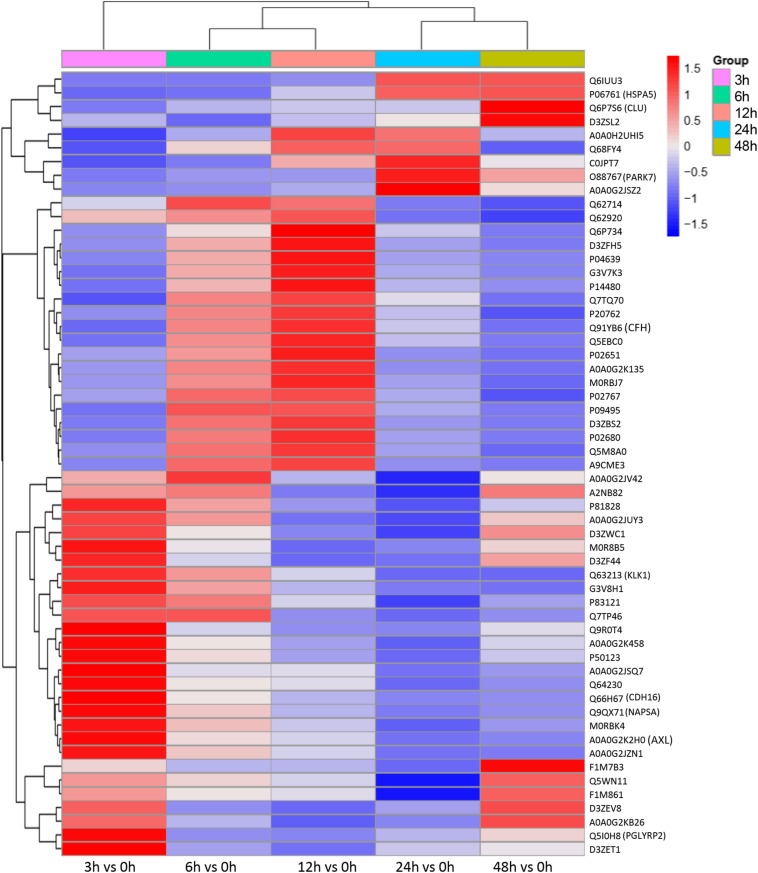
Differentially expressed proteins with a continuous change, which began from the onset of kidney injury and lasted for the whole course of the disease, were sorted, and a heatmap showed the relative expression of these proteins in the indicated time. Protein IDs were showen in the right side of heatmap. *P* < 0.05.

**FIGURE 7:**
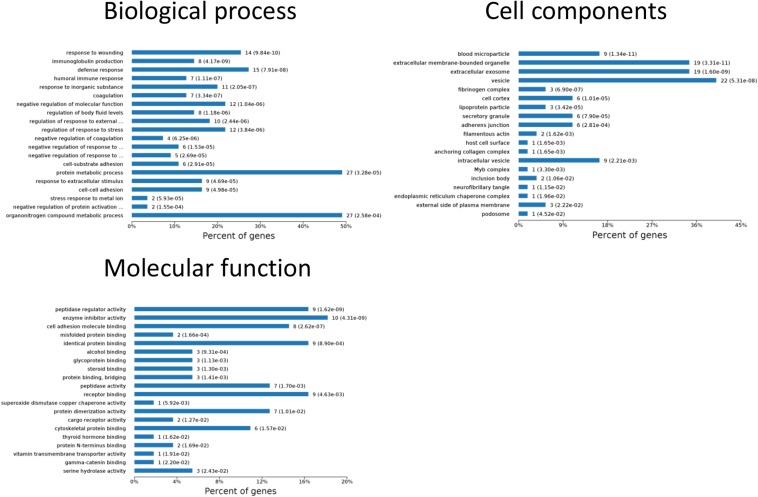
Biological processes, cell components, and molecular functions in which continuously differentially expressed proteins are involved. *P* < 0.05.

To identify the biomarkers used for the early diagnosis of sepsis-induced AKI, we narrowed the category of differentially expressed proteins. Even though the fold change in some proteins was high, the alteration was transient. This kind of protein was not an appropriate candidate to distinguish AKI from the high-risk population. Differentially expressed proteins changing from the onset of kidney injury and lasting the whole course of the disease are ideal biomarkers for early identification and diagnosis. Proteins were sorted according to four kinds of continuous changing models: increase or decrease from the onset of the kidney to 48 h all the time; increase to the peak and then decrease (higher than the expression at 0 h); decrease to baseline levels first and then increase (lower than the expression at 0 h) (Figure 6). The fold change of differentially expressed protein is shown in Supplementary Table S3. Bioinformatics analysis demonstrated that the immunoregulatory biological processes, including metabolic process and response to wounding, were most significantly changed. The top three different cell components remained the same after narrowing the range via a changing trend compared without sorting (Figure 7).

### Diagnostic Accuracy of the Selected Proteins as Sepsis-Induced AKI Fingerprints in Sepsis Human Patients

**FIGURE 8:**
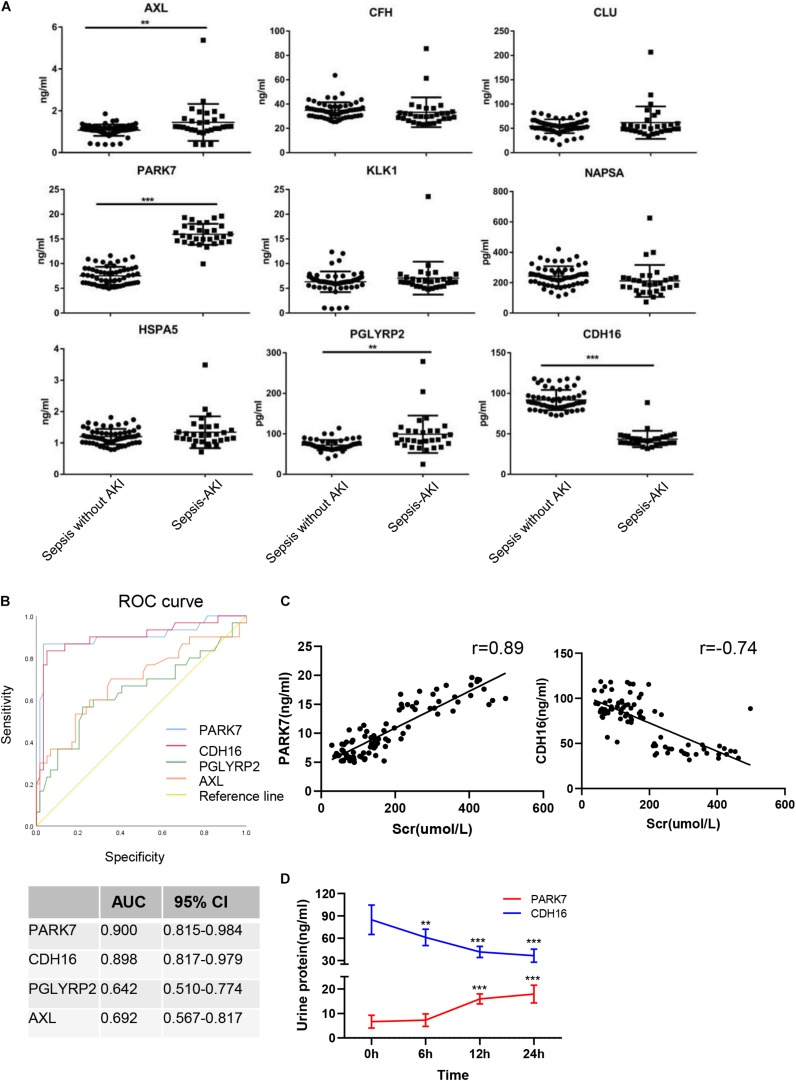
Verification of sepsis-induced AKI-specific biomarkers in sepsis human patients. **(A)** The concentration of nine proteins was validated by ELISA in urine from sepsis-induced AKI patients and non-sepsis-induced AKI patients. ^∗^*P* < 0.05; two-tailed unpaired Student’s *t*-test. **(B)** Validation of 4 differentially expressed proteins by testing their performance in differentiating injury specificity by AUC for PARK7, PGLYRP2, AXL, and CDH16. **(C)** Pearson correlation analysis was performed between expression of PARK7 and CDH16 and Scr. Both PARK7 and CDH16 were correlated to Scr. *P* < 0.01; **(D)** The protein expression of PARK7 and CDH16 in urine from ten patients were detected by Elisa kit at 0, 6, 12, and 24 h after diagnosis with sepsis. **P < 0.01; ***P < 0.001;Compared with 0 h.

The continuously changing differentially expressed proteins associated with inflammation, cell death, proliferation, protein transport, and bacterial hydrolysis were selected as candidate biomarkers. After searching the biological function, nine biomarkers (AXL, CFH, CLU, PARK7, KLK1, NAPSA, HSPA5, PGLYRP2, and CDH16) were selected as potential biomarkers. We tested them in the urine from patients with sepsis-induced AKI (*n* = 30) and sepsis without AKI (*n* = 59). Unpaired *t*-tests showed that in sepsis-induced AKI patients, CDH16 was 0.475-fold of that in non-sepsis AKI patients (*P* < 0.01). AXL, PARK7, and PGLYRP2 were increased significantly (1. 35-, 2. 12-, and 1.37-fold, respectively) in sepsis-induced AKI patients compared with that in the control group (Figure 8A).

To evaluate the usefulness of the selected proteins in discriminating sepsis-induced AKI patients from sepsis, we performed ROC curve analysis. The expression of AXL in human urine was contrary to that in rats after CLP, suggesting that AXL may not be a proper biomarker. ROC curve analysis revealed that proteins (PARK7 and CDH16) could accurately discern sepsis-induced AKI patients, with the AUCs being 0.900 and 0.898, respectively (Figure 8B). AUC analysis revealed that the cutoff values of PARK7 and CDH16 for identifying patients were 13.557 ng/ml (sensitivity 86.7%, specificity 96.6%), and 53.29 pg/ml (sensitivity 83.3%, specificity 94.9%), respectively. We analyzed the correlation between expression of PARK7 and CDH16 and Scr (Figure 8C). We found that PARK7 correlated to Scr positively, while CDH16 was negatively. The *r*-value between PARK7 and Scr was 0.89, and the *r*-value of CDH16 was −0.74. The expression of PARK7 and CDH16 were detected at multiple time point. They were changed in a continuous manner (Figure 8D). These results suggest the high diagnostic potential of these two proteins in distinguishing sepsis-induced AKI individuals from sepsis without AKI controls.

## Discussion

Sepsis is a type of life-threatening organ dysfunction caused by dysregulation of the host response to infection (Coopersmith et al., 2018; Kushimoto et al., 2019). Discovery of biomarkers for sepsis-induced AKI could contribute to early diagnosis of the disease (Peerapornratana et al., 2019). This study identified a set of 56 statistically relevant upregulated and 67 downregulated proteins from rats with CLP-induced AKI using MS. These proteins were found to participate in many biological processes, including complement and coagulation cascades, metabolic pathways, protein processing in the endoplasmic reticulum, and apoptosis. After further detection of differentially expressed proteins in human urine samples, we found that PARK7, and CDH16 are promising candidate biomarkers.

The KDIGO guidelines highlight early AKI diagnosis and treatment, and serum creatinine levels are used as diagnostic criteria (Thomas et al., 2015). Although the serum creatinine test is a practical clinical tool, renal compensation may lead to a lag in the creatinine increase; moreover, 50% of kidney injury cases occur without increases in creatinine (Bellomo et al., 2004; Mishra et al., 2005), resulting in delayed diagnosis and treatment (Alge and Arthur, 2015). Creatinine may also increase due to renal hypoperfusion caused by prerenal factors despite a lack of injury to the renal parenchyma (Ichai et al., 2016). The effectiveness of interventions for kidney injury is largely limited by a lack of sensitivity and by the specificity of diagnostic, therapeutic, and prognostic biomarkers. Biomarker discovery and validation are intrinsically difficult processes because of issues including the emergence of new detection methods, the heterogeneity of patients, difficulties in data analysis, and other physiological confounders (Sarwal et al., 2011). However, proteomics has become a powerful tool for novel biomarker discovery in kidney disease. Novel proteomic approaches can enable discovery of biomarkers suitable for earlier and more accurate diagnosis of renal pathology than traditional biomarkers, such as creatinine and urine proteins. Biomarkers specific to certain phenotypes are robust, stable, and easily accessible by urine testing, thus possessing immense clinical value and providing critical insight for the advancement of AKI therapy (Sigdel et al., 2016). It was found that TIMP2 could ameliorate LPS-induced cytokine release, apoptosis, and cell injury (Li et al., 2019). Biomarker assessment will also contribute to the mitigation of sepsis-induced kidney injury and will help guide optimal renal replacement therapy dosing.

Label-free quantification is a method used in MS that aims to determine the relative amount of proteins (Moore et al., 2004). The use of label-free MS to examine differentially expressed proteins in urine samples largely eliminates the variations and biases in replicate MS measurements (Griffin et al., 2010) and has allowed for the exploration of the mechanism of AKI and the discovery of biomarkers. In our MS results, we found that 123 proteins changed in urine after sepsis-induced AKI. GO analysis revealed that the protein metabolic process was the first altered in the early stage of sepsis-induced AKI. The increase in total cellular metabolism was to provide the energy needed to sustain immune system activation (Pravda, 2014). Mitochondria-centered structural and metabolic alterations occur prior to the onset of AKI, supporting a causative, pathogenic role of mitochondrial damage in kidney injury (Parikh et al., 2015). Possible molecular mechanisms that stimulate the hypermetabolic state include altered mitochondrial bioenergetics, impaired peroxisomal catalase activity and fatty acid oxidation (Vasko et al., 2013). Six hours after induction of AKI, responses to organic substances, wounding and stimuli were the leading biological processes. In the cell component category, extracellular exosomes and vesicles were increased continuously from 6 to 48 h. Exosomes, a type of nanoparticle, function as intercellular shuttles that bud from the host cell membrane and carry information to recipient cells (Barile and Vassalli, 2017). Exosomes can be transferred in biological fluids to interact with remote organs and can be secreted in urine (Karimi et al., 2018). In the KEGG analysis, the immune system was in the center place of AKI. The host defense against infection is an adaptive response to decrease the pathogen load, limit tissue injury, and restore homeostasis (Gomez et al., 2017).

To identify the biomarkers used for the early diagnosis of sepsis-induced AKI, we narrowed the category of differentially expressed proteins by changing their trend to avoid transient alterations. Proteins with stable and continuous changes are ideal biomarkers for the early identification and diagnosis of AKI. Even when proteins were sorted by changing trends, the biological process and cell components remained quite consistent. Here, we used ELISA to identify panels of proteins that may be used as fingerprints of sepsis-induced AKI. Nine proteins were detected in human urine with sepsis-induced patients. AXL, CDH16, PARK7, and PGLYRP2 were significantly changed in the urine of patients suffering from sepsis-induced AKI. AXL regulates oxidative stress and inflammation by including nuclear factor-kappa B (NF-κB). The previous study identifies that AXL is a promising therapeutic target for the prevention of AKI-to-CKD transition (Chen et al., 2019). However, the expression of AXL in the rat’s urine was decreased after sepsis. The possible explanation was that the patients always suffered from multiple diseases, and the source of infection was not limited to abdominal bacterial infections. PARK7 acts as a positive regulator of androgen receptor-dependent transcription and functions as an inhibitor of cellular oxidative stress and a regulator of mitochondrial function, autophagy, and apoptosis (Oh and Mouradian, 2017). The protective effect of PARK7 signaling was identified in renal tubular epithelial cell injury induced by ischemia (Yin et al., 2019). PGLYRP2 plays a scavenger role by digesting biologically active peptidoglycan into biologically inactive fragments. CDH16 is a member of the cadherin superfamily, calcium-dependent, membrane-associated glycoproteins. Expression is exclusively in the kidney, where the protein functions as the principal mediator of homotypic cellular recognition, playing a role in the morphogenic direction of tissue development. As PARK7, PGLYRP2, AXL, and CDH16 were significantly changed and CDH16 is a kidney-specific protein, we used PARK7, PGLYRP2, AXL, and CDH16 to perform ROC curve analysis. The diagnostic sensitivity and specificity of PARK7 and CDH16 were higher than those of neutrophil gelatinase-associated lipocalin (NGAL), which was used to diagnose AKI, particularly infection-mediated AKI (Kim et al., 2016). PARK7 and CDH16 were changed continuously, which were suitable biomarkers for early detection.

There are some limitations of the present study that should be clearly addressed. First, the expression of proteins cannot be matched with the extent of the kidney injury. We used a standardized approach to induce sepsis and to obtain stable mortality rates. However, the extent of AKI was different, and the urine output of each rat was so scarce that the urine from five rats was pooled together subjected to MS. Second, it would be helpful to study the course of PARK7 and CDH16 expression in both serum and urine at specific time points. *In vitro* and *ex vivo* experiments are needed to confirm the biological function of these biomarkers. Clinical trials evaluating diagnosis and management should focus on precise populations and consider biomarker enrichment strategies (Kellum, 2016). Furthermore, to ensure the sensitivity and specificity of biomarkers, a combination of multiple proteomics analysis of samples from urine, serum, and tissue will be the future direction.

## Conclusion

In summary, we performed proteomics at multiple time points to identify altered proteins in urine and understood the biological process in sepsis-induced AKI. We analyzed urine proteins with spatiotemporal dynamics to sensitively monitor biological alterations in sepsis-induced AKI, and two distinctive biomarkers were defined in patients with sepsis-induced AKI. The results obtained with our systems biology approach might provide a theoretical basis for further research to unravel the molecular mechanisms and to develop novel biomarkers for sepsis-induced AKI.

## Data Availability Statement

All included data are available in the public domain, and all references are included in our reference list. Extracted data and calculations will be made available to individual scientists upon reasonable request.

## Ethics Statement

The animal study was reviewed and approved by the Animal Care and Use Committee of Nanchang University. The studies involving human participants were reviewed and approved by the Research Ethics Committee at the Zhongnan Hospital of Wuhan University. The patients/participants provided their written informed consent to participate in this study.

## Author Contributions

YL conceived the project, designed the project, extracted and analyzed the data, drafted the manuscript, and approved the final manuscript. JL revised the manuscript and approved the final manuscript. JC contributed to the CLP model and urine collection. YQ collected patients’ urine samples. JZ and YZ drafted the part of discussion and background of the manuscript. YL, JC, and JZ conducted the experiments. FL and ZP designed the project, edited the manuscript, and approved the final version.

## Conflict of Interest

The authors declare that the research was conducted in the absence of any commercial or financial relationships that could be construed as a potential conflict of interest.

## Supplementary Material

FIGURE S1 Analysis of data distribution and quantitative abundance. **(A)** Analysis of data distribution. The histogram represents the data distribution of each sample. Experimental data basically conformed to the normal distribution, which could be used for subsequent statistical analysis. **(B)** Analysis of the quantitative abundance of protein. The abscissa represents the sample name, and the ordinate represents the protein classification (normalized results). Each point in the figure represents the abundance value of a protein. A polyvine diagram was formed by connecting the points. The black box in the middle was the boxplot of the protein abundance of each sample. The orange polyline represents the abundance distribution trend of the protein P3052 in different samples.

FIGURES S2–S4 The biological process, cell components, and molecular function of differentially expressed proteins in which differentially expressed proteins in urine at the indicated time (3, 6, 12, 24, and 48 h) were compared with those at 0 h. *P* < 0.05.

TABLE S1 Concentration of serum creatinine in CLP-induced AKI (μmol/l).

TABLE S2 One-way ANOVA was performed to analyze the differential expression protein in the urine after sepsis-induced AKI. *P* < 0.05.

TABLE S3 The continuous changing protein in the urine after sepsis-induced AKI. *P* < 0.05.

## Abbreviations

AKI: acute kidney injury
AUC: area under curve
CLP: cecal ligation and puncture
DAMPs: damage-associated molecular patterns
KDIGO: kidney disease improving global outcomes
KEGG: kyoto encyclopedia of genes and genomes
LC-MS: liquid chromatography–mass spectrometry
MS: mass spectrometry
NF- κ B: nuclear factor-kappa B
NGAL: neutrophil gelatinase-associated lipocalin
PAMPs: pathogen-associated molecular patterns
PCA: principal components analysis
PPI: protein-protein interactions
ROC: receiver operating characteristic.
