# Supplementary material for: Analysis of Spatiotemporal Urine Protein Dynamics to Identify New Biomarkers for Sepsis-Induced Acute Kidney Injury
Source: Front Physiol. 2020 Mar 3;11:139. doi: 10.3389/fphys.2020.00139 (PMC7063463; doi:10.3389/fphys.2020.00139)

Supplementary Figure 1

A

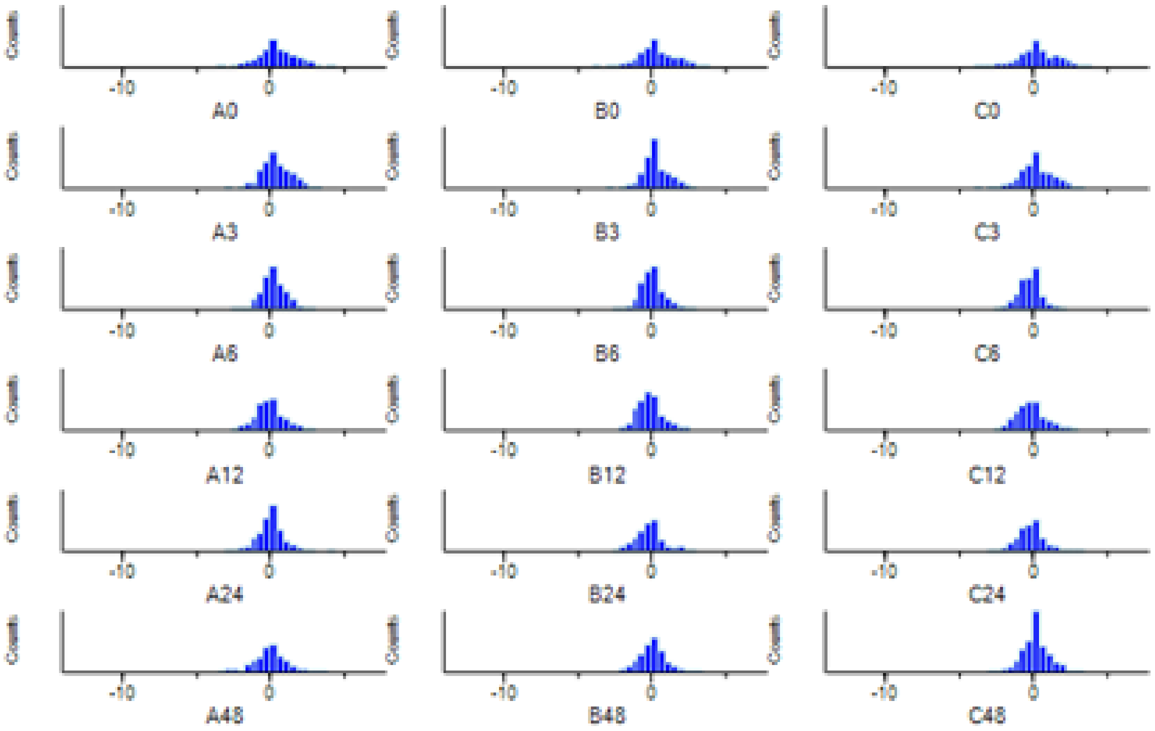

B

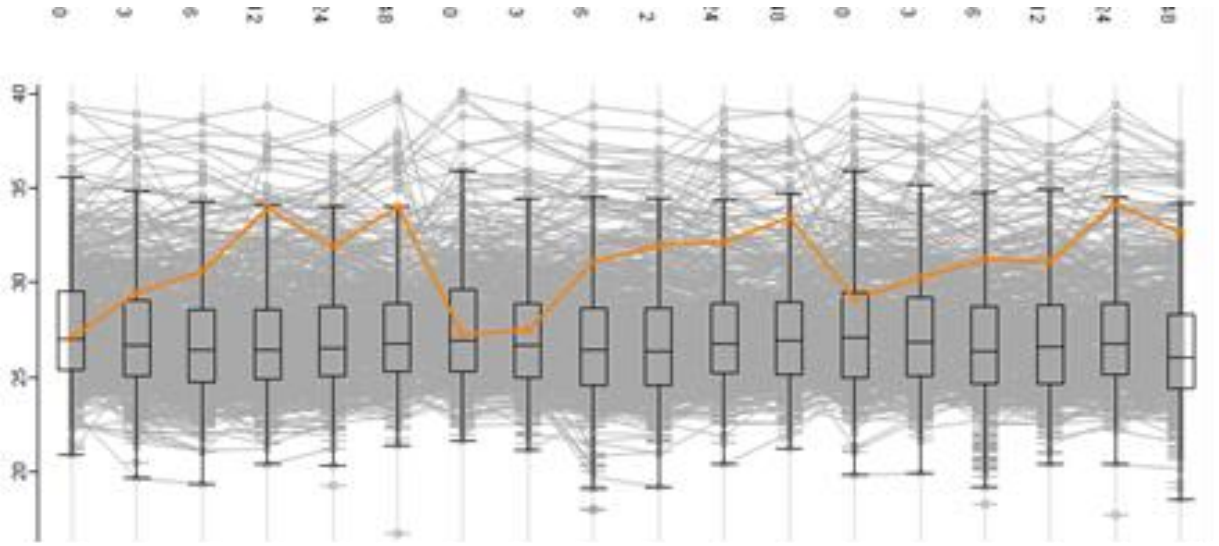

Supplementary Figure 2

Biological process

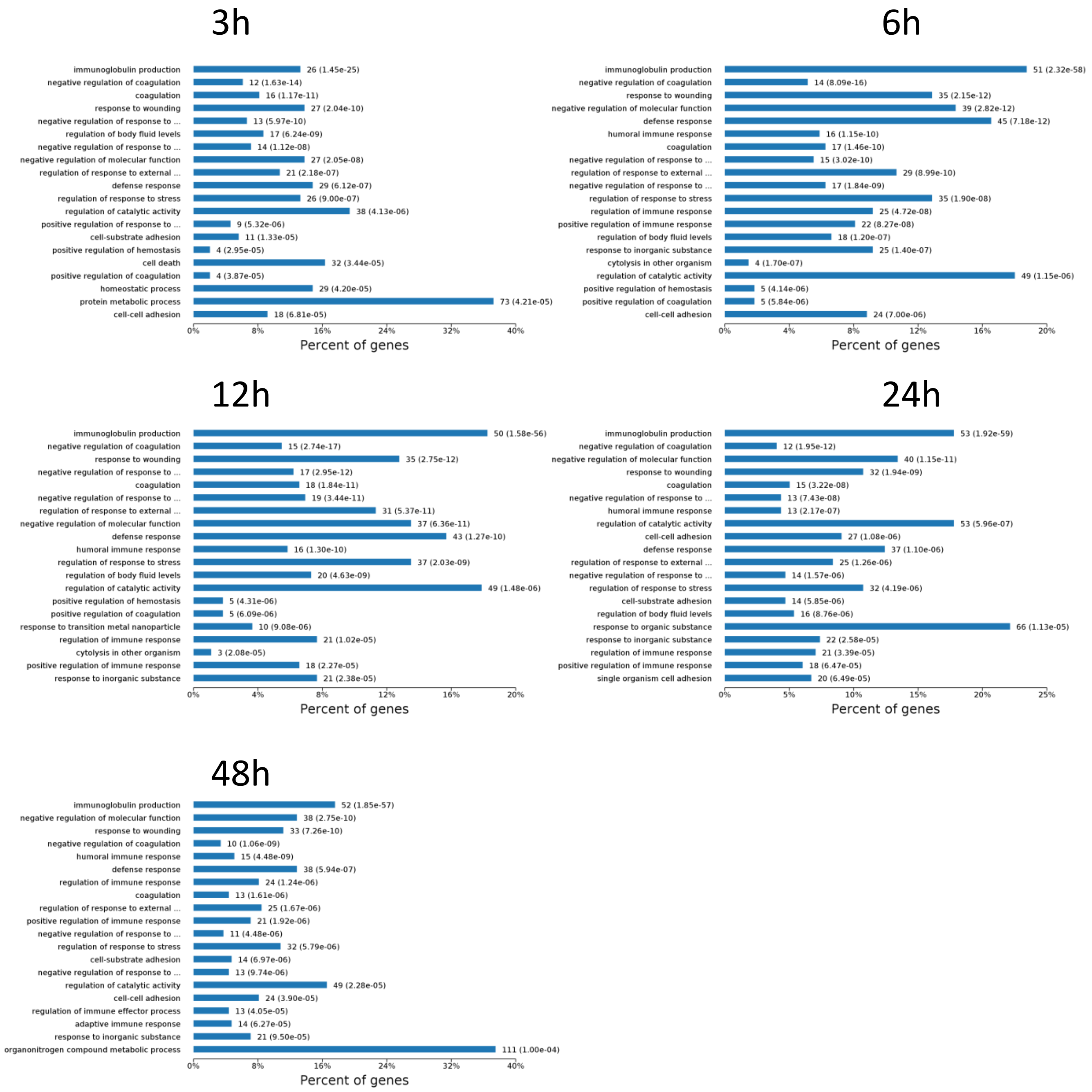

Supplementary Figure 3

Cell components

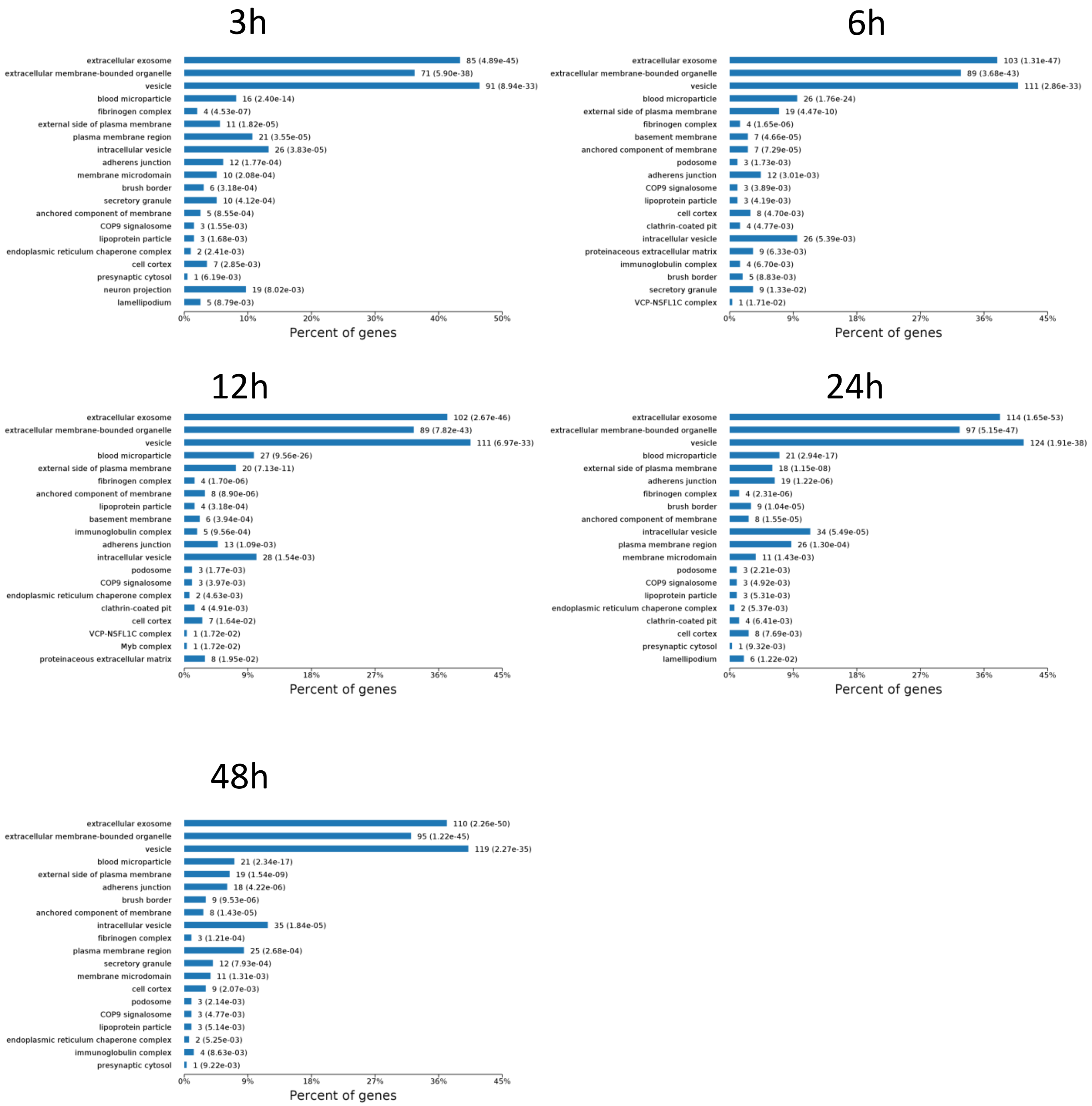

Supplementary Figure 4

Molecular function

3h

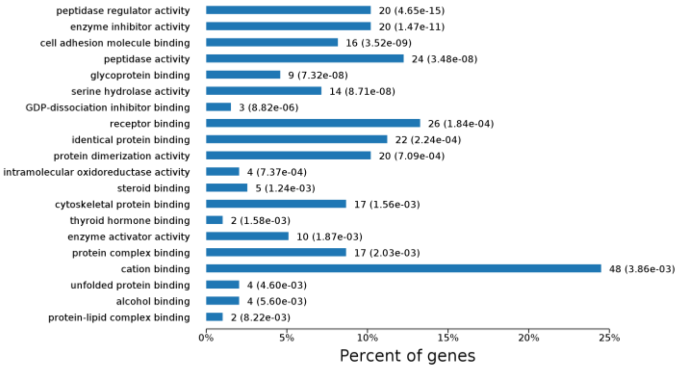

6h

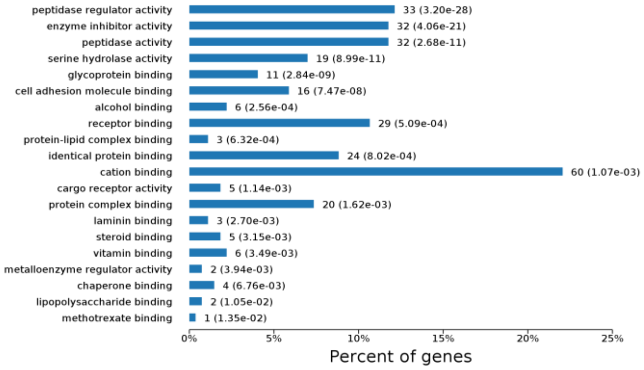

12h

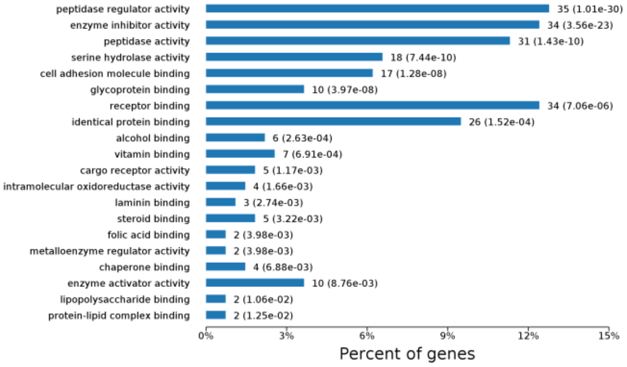

24h

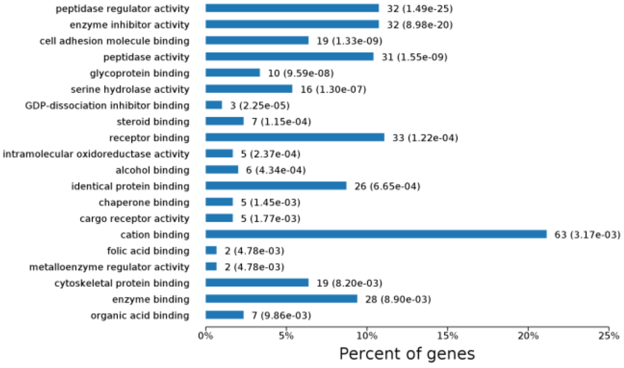

48h

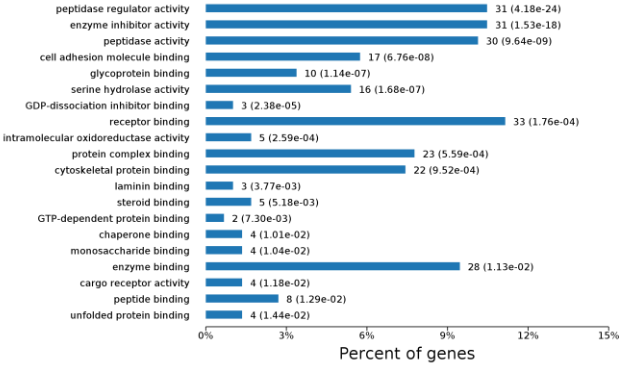

Supplement: FIGURE S1 — Analysis of data distribution and quantitative abundance. (A) Analysis of data distribution. The histogram represents the data distribution of each sample. Experimental data basically conformed to the normal distribution, which could be used for subsequent statistical analysis. (B) Analysis of the quantitative abundance of protein. The abscissa represents the sample name, and the ordinate represents the protein classification (normalized results). Each point in the figure represents the abundance value of a protein. A polyvine diagram was formed by connecting the points. The black box in the middle was the boxplot of the protein abundance of each sample. The orange polyline represents the abundance distribution trend of the protein P3052 in different samples. [file Data_Sheet_1.PDF]
